# Evaluation of Aerosol Electrospray Analysis of Metal-on-Metal Wear Particles from Simulated Total Joint Replacement

**DOI:** 10.3390/s19173751

**Published:** 2019-08-30

**Authors:** Gobert von Skrbensky, Karoline Mühlbacher, Emir Benca, Alexander Kolb, Reinhard Windhager, Georg Reischl, Georg Reinisch

**Affiliations:** 1 Department of Orthopedics and Trauma Surgery, Medical University of Vienna, Währinger Gürtel 18-20, 1090 Vienna, Austria; 2 Faculty of Physics, University of Vienna, Strudelhofgasse 4, 1090 Vienna, Austria; 3 Ingenieurbüro für technische Physik, Speichmühlengasse 1, 2380 Perchtoldsdorf, Austria; 4 Biomechanische Forschungs-Gesellschaft m.b.H., 1040 Vienna, Austria

**Keywords:** debris, particles, wear, implants, prosthesis

## Abstract

Wear is a common cause for aseptic loosening in artificial joints. The purpose of this study was to develop an automated diagnostical method for identification of the number and size distribution of wear debris. For this purpose, metal debris samples were extracted from a hip simulator and then analyzed by the electrospray method combined with a differential mobility analyzer, allowing particle detection ranging from several nanometers up to 1 µm. Wear particles were identified with a characteristic peak at 15 nm. The electrospray setup was successfully used and validated for the first time to characterize wear debris from simulated total joint replacement. The advantages of this diagnostic method are its time- and financial efficiency and its suitability for testing of different materials.

## 1. Introduction

Aseptic loosening is a prevalent complication of major arthroplasties and it is the most common cause of hip and knee replacement [1,2,3,4]. Previous reports show that up to 70% [5,6,7] of all revisions of the hip and up to 44% [4,8,9] of the knee joint can be traced back to aseptic loosening. Revision surgeries often show inferior results compared to primary intervention. They are costly, time-consuming, bear risk, and are stressful for the patient.

Wear is commonly regarded as a possible source for aseptic loosening in artificial joints and can be found as polyethylene, metal, ceramic, and even cement particles, depending on the used implant material(s). Moreover, an influence on the biological activity of osteoblasts and osteoclasts has been shown, caused by different number and size of particles, especially of metal wear particles [10,11,12,13,14]. Thus, the characterization of wear particles in terms of number and size is essential for the study of their biological effects on cells and tissue [2,13,15,16,17,18,19].

As metal wear particles are commonly smaller than polyethylene (PE) particles, they show a potential to induce cytokine release from macrophages leading to bone loss and aseptic loosening [1,2,20].

Previous reports have analyzed metal wear particles isolated from periprosthetic tissue, synovial fluid [21] or fluids from joint simulator tests [22,23,24,25,26]. These particles were characterized under electron microscopy [12,20,26,27,28] using manual or semi-automated counts.

Automated methods using cell counters, like those presented by Malony et al. [29] and Mochida et al. [30], seem to underestimate the number of nanometer-sized particles [31]. Currently published by different study groups the smallest particles analyzed (UHMWPE, ceramic and metal) range down to 10 nm [31,32]. The relevance of analyzing particles of this size range was attributed by the introduction of an index for functional and specific biological activity [26,33]. The authors of the present study aimed to introduce an automated method for wear particle characterization within a range of 5 nm–1 µm: the electrospray method. This method has been previously successfully applied in the characterization of motor vehicle originated emissions [34], and is well established in biological and physical research, e.g., nanotechnology, immunology, mass spectrometry, etc. In aerosol physics in particular, the electrospray a very common tool and used as a device that employs electricity to disperse liquid into a fine aerosol. This device uses the electrospray ionization (ESI) technique, used in mass spectrometry to produce ions. The electrospray ionization was originally developed for the analysis of biological macromolecules by John Bennett Fenn in 1989 [35]. One application of this method is the determination of the number and size of submicron particles. A further advantage of this method is its independence of material in use.

To the knowledge of the authors, the method of electrospray method has not been used previously to characterize wear debris from total joint replacement.

## 2. Materials and Methods

The source of metallic debris under investigation is the articulation surface of a low carbon metal-metal bearing combination and was generated in a hip simulator. The matching bearing partners were fabricated by a single manufacturer (Sikomet; Plus Endoprothetik AG, Rotkreuz, Switzerland). All femoral heads (diameter: 28 mm) were manufactured from a cobalt-28chromium-6molybdenum alloy (CoCrMo) forging to meet the requirements of the ASTM standard F799. The alloy is obtained by vacuum melting and has an especially fine structure with almost no carbides [36].

The inserts were of a sandwich design, with a polyethylene interface layer (UHMWPE RCH-1000 to meet the requirements of the ISO standard 5834/1/2) and a bearing surface of the CoCrMo alloy described above. The inserts were fixed in a cementless self-tapping adaptor. All components were fabricated by the same manufacturer. The 28 mm metal-on-metal bearing systems were placed into an anatomically correct hip simulator, E-SIM (Biomechanische Forschungs-Gesellschaft m.b.H, Vienna, Austria) [37,38]. The tests were each run for 5 million cycles according to ISO 14242-1. In brief, the hip joint is aligned in the anatomically correct position with three axial displacements (flexion/extension, abduction, adduction, and internal-/external rotation. The joint was loaded with the double-peak load regime up to three kN. [39] The 28 mm femoral ball head and acetabular cup were immersed in absolute Ethanol (nano-grade) (Merck, Darmstadt, Germany). The simulator was run for five million cycles without stopping and the lubricant containing the wear particles was collected at the end of the simulator test. Another test was also run for 5 million cycles in calf serum lubricant (PAA, Pasching, Austria) diluted to a protein concentration of approximately. 30 g/L without additives with intervals for cleaning and reassembling according to the protocol of the ISO standard was used. Lubricant samples were collected after the running-in phase between 2 and 5 million cycles. For each test run a new sealed bearing system was used. 

For validation of the measurement setup reference silica spheres with a nominal diameter of 102 nm (Thermo Scientific, Fremont, CA, USA) were used.

### 2.1. Preparation of the Samples

Samples were taken from the hip simulator test runs using calf serum and nano-grade ethanol (Ethanol) as a lubricant between the articulating partners of the bearing surfaces. In order to eliminate all organic molecules, the hip simulator test run was performed using pure Ethanol. The calf serum lubricants were digested following the alkaline digestion protocol of Catelas et al. [23]. Although the tribological behavior of ethanol and synovial fluid significantly differ, the use of ethanol was specifically chosen in this study to evaluate the electrospray method in test runs with the calf serum. The use of ethanol is clearly not representative of synovial samples in clinical routine. However, to establish a physical relevant baseline for the electrospray method, it was necessary to use an ultra-pure test liquid. 

All fluid samples from the simulator test-runs were stored at 5 °C until preparation. The original samples were then divided into several samples with different concentrations. The samples were agitated and the fluid was removed by a sterilized syringe. Besides a pure fluid sample, following sample concentrations of the simulator test-run were produced: 1:100, 1:25, 1:5, 1:4, 1:3. The original fluid was diluted with pure Ethanol and the specimens were stored in centrifuge vials. To reduce agglomerates the fluid was agitated in a commercial ultra-sonic bath for 2 h before being tested. 

### 2.2. Electrospray Method

**Figure 1:**
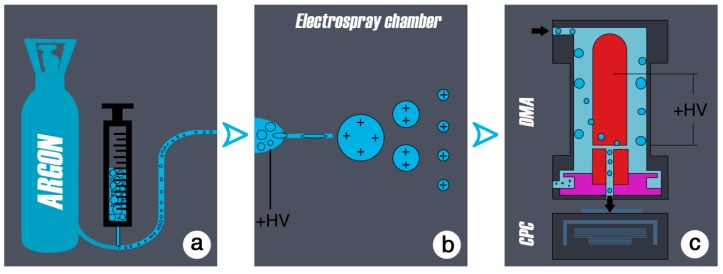
(**a**) A vial containing the liquid sample (e.g., calf serum) is attached to the electrospray. Argon gas applies pressure in order to push the liquid from the vial into the electrospray chamber. A quartz capillary connects the vial to the electrospray chamber where high voltage is applied. (**b**) High voltage (+HV) in the electrospray chamber forces the entering liquid to produce a Taylor cone (when the liquid enters the chamber and is exposed to the high voltage, the forces of this electric field force the liquid to form a cone with convex sites and a rounded tip). Droplets emerging from the tip of the cone carry a large number of charges, however when they dry out (due to the pure-air environment in the chamber), the charge density rises which leads to Coulomb explosion. Single particles carrying a single charge are the result. (**c**) The particles are introduced into the differential mobility analyzer (DMA). By varying the voltage, only the corresponding particle size with a well-defined electric mobility will exit the DMA and will be measured. After leaving the DMA, particles are counted by a condensation particle counter (CPC) and further analyzed by control software. The output is number-size distributions shown in this paper.

A schematic process flow of the aerosol method is presented in Figure 1.

The electrospray method was originally developed by John Bennett Fenn in 1989, for analysis of biological macromolecules (proteins in particular) [35]. It is well established in biological and physical research (nanotechnology, immunology, mass spectrometry, to produce ion sources to test emissions, e.g., in the motor vehicle industry etc.) for the generation of well-defined particles ranging in size from a few nanometers to single molecules. In aerosol physics, the electrospray is a common tool for producing fine aerosols from any kind of liquid. An aerosol is a colloid of solid or liquid particles in a carrier gas. The liquid of interest is contained in a vial, which is attached to the electrospray-chamber.

**Figure 2:**
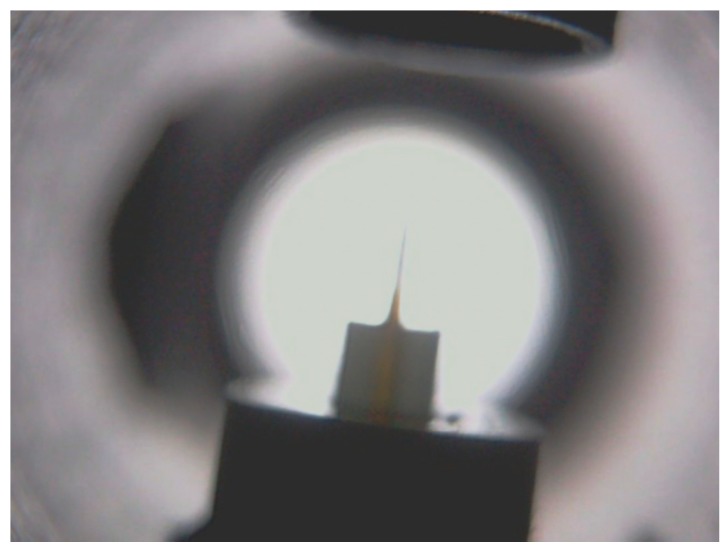
Inside of the electrospray chamber. The tip of the quarz-capillary is seen in the center.

**Figure 3:**
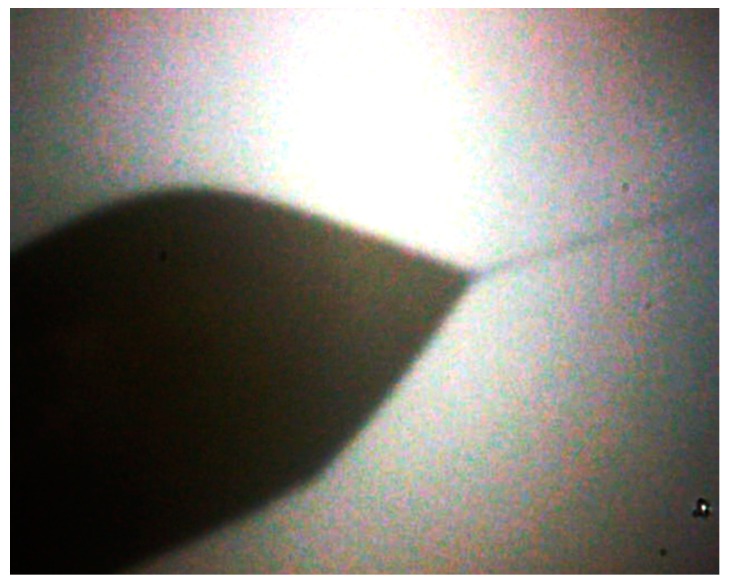
Formation of the Taylor cone.

To transform the emulsion of particles into the aerosol phase, the diluted samples in this study were sprayed by an electrospray device. The liquid sample was pressed by a purified air/argon gas through a quartz capillary of 50 µm inside diameter. When leaving the capillary the liquid was exposed to a high electric field (10^7^ V/cm) (Figure 2 and Figure 3). This charged the droplets and a so-called Taylor cone [40] is initiated. The highly charged droplets emitted from the tip of the Taylor cone (with a diameter of about 50–100 nm) are further dispersed by a coulomb explosion during the drying process so that ideally every drop contained a single particle. This resulted in an aerosol of wear debris particles that could be analyzed at this point with standard aerosol characterizing methods like the electromobility spectrometry.

The particles were then given a well-defined charge using a neutralizer. The neutralized aerosol was passed through a differential mobility analyzer (DMA) to determine the size of the particles. The DMA is a cylindrical capacitor; it selects particles of a certain electrical mobility, which depends on particle size and charging state. The particles were introduced to the DMA to its outer electrode. The charged particles migrated, driven by the electric field and according to the electrical mobility from the outer electrode of the capacitor through a clean air shield region towards the inner electrode, where they entered the condensation particle counter (CPC). The condensation particle counter determines the concentration of the extracted mobility fraction [27,41]. In the CPC, the aerosol was passed through a saturated butanol environment. The saturated buthanol vapor containing the aerosol particles was introduced into a cooler, where the super-saturated vapor has condensed onto the particles. This resulted in droplets of several microns in size, which could be counted individually by light scattering. Varying through a range of field strengths in the DMA gave finally a detailed size distribution for the wear particles. However, any impurities contained in the solution droplets produced particles after being dried. This background could not be completely avoided, but minimized by using pre-cleaned solutions and clean solvents (i.e., spectrograde ethanol). The electromobility method can be used to determine the size of particles ranging from 4.5 nm to hundreds of nanometers in diameter.

The size distribution obtained by the DMA provided the number concentration of particles versus the mobility equivalent diameter. To cover the full-size range of wear debris particles, a DMA with a 4.5–200 nm size range (the lower size limit is given by the CPC in use) was used. However, also a DMA with a larger size range was used to exclude the existence of particles larger in size.

In order to characterize particles with scanning electron microscopy (SEM), a 0.2 µm filter (Isopore Membrane Filters 0.2 GTTP, Millipore, Billerica, MA, USA) was inserted between the electrospray setup and the DMA. 

The presented setup used a neutralizer between the electrospray and the DMA since the aerosol particles generated by the electrospray method might have carried more than one elementary charge. The neutralizer served to provide a well-known charging state on the aerosol particles, a necessary condition for the electromobility spectrometry. This was achieved by submitting the aerosol to a bipolar ionic atmosphere for a certain amount of time. This atmosphere was produced by an alpha source (^241^Am—Americium in this case).

## 3. Results

**Figure 4:**
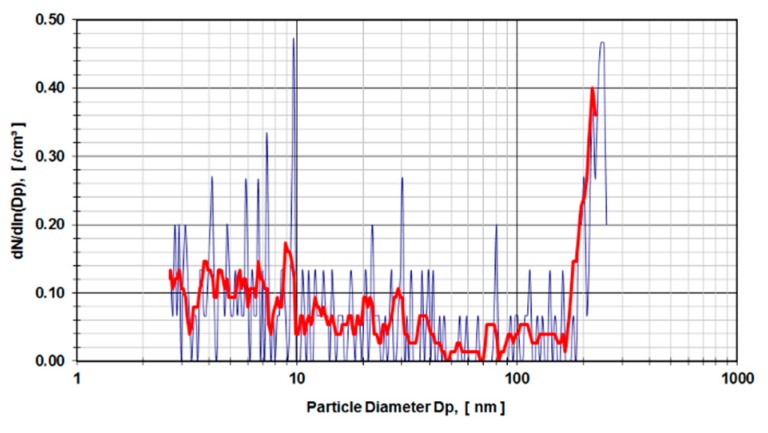
Metal-on-metal articulation in digested calf serum, dilution 1:3.

**Figure 5:**
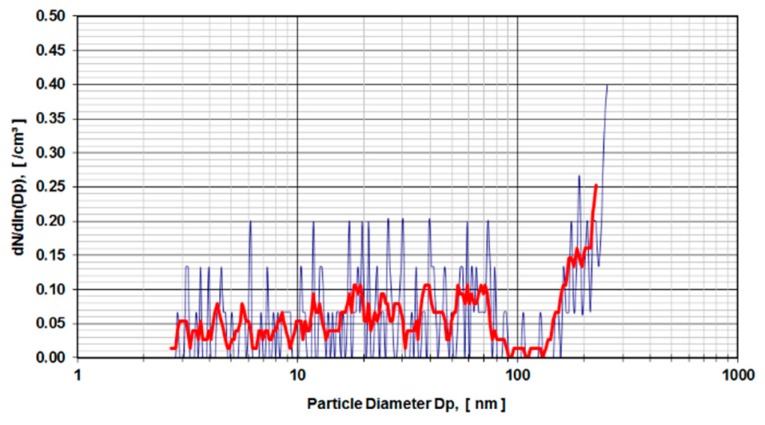
Calf serum without metal-on-metal articulation, digested, dilution 1:4.

The results are presented as particle diameter (D_p_) (nm) versus concentration density $\frac{dN}{d\ln(D_{p})}$ in (/cm^3^). The bold red line in Figure 4 and Figure 5 show the arithmetic mean of 5 individual test runs.

Digested calf serum samples (sample V3) of a metal-on-metal hip simulator articulation were used in the first run of experiments. The samples V3 were diluted 1:3 and 1:4 and exposed to a DMA. Figure 4 presents the distribution of particle sizes within the range of 4.5–250 nm for a dilution of 1:3. Calf serum, which was not used in a hip simulator test run but stored for an equivalent amount of time under the same environmental conditions, was digested and diluted 1:4 (Figure 5) and used as a control sample. The observed peaks were caused by organic molecular clusters in the calf-serum.

The measurements of the CPC were interjected in order to insert the filters into the aerosol line. The filters were loaded with the aerosol containing the particles of the digested calf serum experiments and exposed to scanning electron microscopy (Zeiss Supra 55 VP, Oberkochen, Germany). The surface of the filters was inspected using 5000 and 20,000 magnification. The material consistency of the particles was analyzed using an EDX detector attached to the SEM (EDX from Oxford Instruments, Abingdon, UK). Several areas containing particles were examined. However, no traces of the cobalt, chromium or molybdenum were found. The reason, therefore, is the high density of organic residues, which dominate the SEM spectrum.

**Figure 6:**
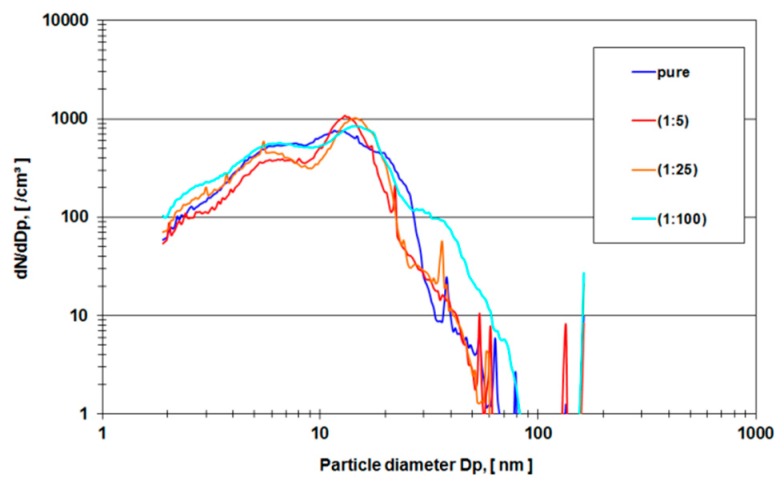
Ethanol probe, metal-on-metal, dilution 1:5, 1:25, 1:100 and pure.

**Figure 7:**
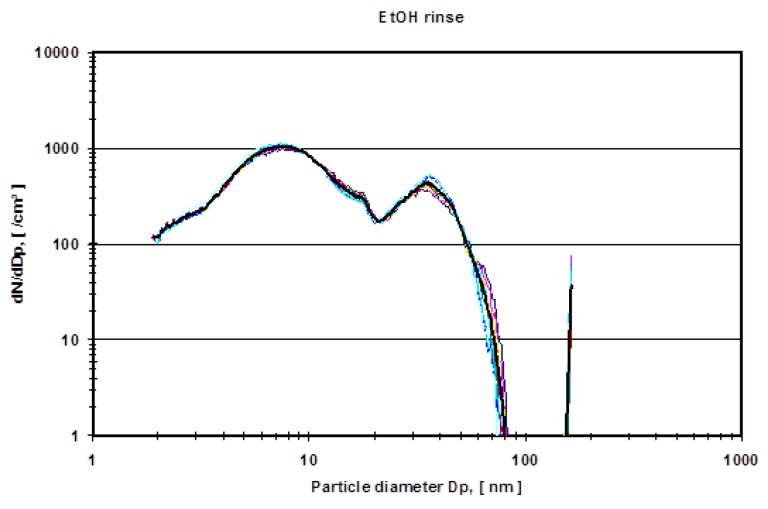
Ethanol rinse, pure ethanol, no dilution.

In order to avoid organic background and to verify the experimental method, a second run of experiments was carried out using pure ethanol as a lubricant in the metal-on-metal setup of the hip simulator. Two lubricant samples were examined (a) (ethanol sample) the nano-grade ethanol lubricant that was exposed to the metal-on-metal articulation for 5 million cycles with different dilutions (1:5, 1:25, 1:100 and pure) and (b) (ethanol rinse) nano-grade ethanol that was used to rinse the mechanical setup of the hip simulator. Figure 6 depicts a rise of particle concentration with a diameter between 15–20 nm which is not present in the pure rinse of the system. Figure 7 shows the mean particle diameter distribution of the rinse of the system in comparison to the effective ethanol lubricant containing the metal wear particles.

**Figure 8:**
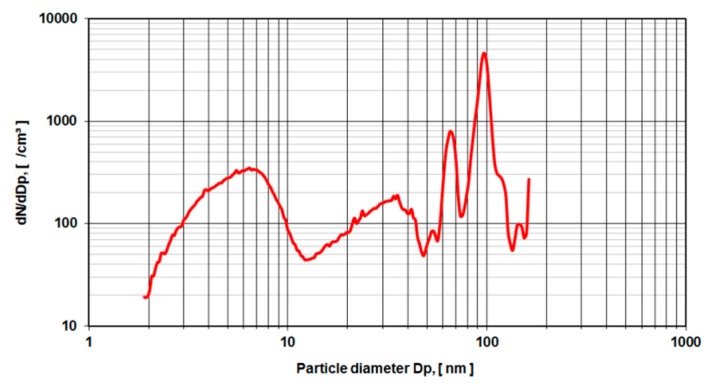
Reference silica spheres with 102 nm nominal diameter, no dilution.

To validate the measurements of metal-on-metal wear particles in nano-grade ethanol NIST-certified polymere reference spheres with a nominal diameter of (102 ± 3 nm) were used in the same electrospray setup (Figure 8). Also, gold reference spheres with a nominal diameter of 10, 20 and 50 nm have been investigated. However, the background caused by the anticoagulant agent could not be suppressed, which resulted in peaks at 8, 35, 65 nm The peaks at 140 nm and over may be attributed to doublets of particle agglomerates.

## 4. Discussion

The size range of wear particles originating from metal-on-metal articulations in joint arthroplasty is reported in the literature from several microns down to the detection limit of analyzing equipment like SEM [19,42,43,44] and TEM [28,42,45]. Various authors reported metal wear particles in retrieved capsular tissue samples from failed metal-on-metal hip articulations in different size ranges [17,18,45,46,47,48,49,50,51] by light microscopy through staining of the tissue samples [52]. Urban et al. [52] identified metal wear particles in the spleen and liver with a size of 0.1–7 µm. Consequently, the elements of the metal bulk material cobalt, chromium, and molybdenum dissolve in the lubricant environment of body fluids. Various authors report the rise of analytical element levels in synovial fluid, blood and urine after implantation of metal-on-metal arthoplasties [53,54,55,56,57].

So far, Brown et al. reported the smallest traceable metal particles from digested tissues and simulator studies with TEM. The authors reported sizes of 6 nm which were still traceable in TEM. The quantification of particles is done semi-automated by particle counts in TEM images [22].

In comparison to the literature, the electrospray method is an automated counting device for particles between the detection limit (several nm) and 300 nm.

As learned from the first series of tests carried out according to ISO 14242-1using 28 mm metal-on-metal hip articulations in a simulator using calf serum as lubricant medium, the results show an evident rise in particle concentration from 160 nm up to the detection limit of 250 nm of the DMA measurement device (Figure 4). In comparison to the control run (calf serum without metal wear particles) with a higher dilution factor (1:4 instead of 1:3), the rise of particle concentration starts at 130 nm (Figure 5). The distribution of particles below 100 nm diameter gives evidence of a stochastic distribution of particles, which might be caused by arbitrary organic molecules that have not been dissolved in the digestion process. Since the rise of a signal is evident for both probes (calf serum containing metal wear debris and calf serum as a control) it is obvious that the detected particles do not originate from metal debris only. The results indicate that the process of digestion does not eliminate organic molecules in that size range (up to 250 nm) adequately. 

As a control specimen, a 0.22 µm filter membrane was inserted into the aerosol line in order to identify and confirm the high density of organic molecules as measured in samples obtained from test runs in calf serum using the electrospray method. Thus particles were collected on the filter and examined by SEM. Both pictures of the SEM indicate the presence of particles of the size of several microns down to 100 nm and presumably less. However, none of the particles detected could be attributed to the metallic elements of the articulating material due to the overwhelming number of organic particle larger in size and number. Spot and areal EDX measurements were conducted on several filter areas with a negative result. 

In order to eliminate these organic molecules, nano-grade ethanol was used as a lubricant in the hip simulator (Figure 6). The total amount of particles rose dramatically (compare the ordinate of Figure 4 and Figure 5 with Figure 6 and Figure 7). For all different dilutions the characteristics, as well as the gross average amount of particles, stay constant with a distinguishable peak at approximately 15 nm. The authors have conducted also several measurements with the electrospray method using pure nano-grade ethanol rinse of the hip simulator, which is depicted in Figure 7. The characteristic of this curve also shows a rise of particle concentration up to 7 nm, a characteristic drop at 20 nm and another local maximum at 40 nm. The authors attribute these particles counts to nonvolatile residues (NVR) in the nano-grade ethanol. Thus a definite, characteristic size range of metallic wear particles in ethanol used as lubricant seems to be near 15 nm since the control measurements indicate no presence of NVR’s in this size range. 

For validation purposes, the same setup of aerosol particle characterization was used with defined silica spheres of 102 nm diameter. These sample probes are produced and tested for calibration purposes and come with a certificate of calibration by the US National Institute of Standards and Technology (NIST). Again, a rise of particle counts is met below 6 nm diameter with another rise up to 20 nm. From there on the curve becomes more characteristic and shows a major peak near 100 nm and two more peaks at 70 nm and 50 nm to the left of the 100 nm peak, and a shoulder at 120 nm and another peak at 140 nm. 

The main peak at 100 nm is attributed to the nominal diameter of the reference spheres of 102 nm diameter. These particles carry a single elementary charge. Particles with two or three elementary charges pass quicker through the electromagnetic field of the electrospray set up and are thus identified as “smaller” particles namely with the size of 70 nm for two elementary charges and 50 nm for three elementary charges. The shoulder at the size of 120 nm remains unclear to the authors. However, the diameter size of 140 nm may be attributed to doublets of particle agglomerates with a resulting equivalent diameter of $\sqrt{2}$ of the original sphere diameter. 

As a result of the data presented, wear particles originating from simulated metal-on-metal articulations are identified with a characteristic peak at 15 nm.

## 5. Conclusions

This study introduces the use of the electrospray method as a potential diagnostic tool for identifying metal debris of artificial joints in number and size. This method has the advantage compared to conventional methods for identifying metal particles, such as SEM, and of being more time and economically efficient. Even though the authors were not able to fully eliminate organic molecules from simulator samples, for which further studies will be required, the method was successfully validated with particles defined in number and size.

The authors believe in the high potential of the evaluated method as an efficient tool in clinical routines.
